# Forearm blood flow and vascular conductance improve after 18 weeks of bouldering training in novice climbers

**DOI:** 10.3389/fspor.2026.1818579

**Published:** 2026-06-01

**Authors:** Julia Maria Rosa da Silva, Sara Sobreviela Sánchez, Ana Júlia Araújo Pucci, Maria Urbana Pinto Brandão Rondon, Guilherme Wesley Peixoto da Fonseca

**Affiliations:** 1 School of Physical Education and Sport, University of São Paulo (EEFE-USP), São Paulo, Brazil; 2 Laboratório de Nutrição e Metabolismo Aplicados à Atividade Motora, EEFE-USP, São Paulo, Brazil; 3 Laboratório de Controle Autonômico da Circulação, EEFE-USP, São Paulo, Brazil

**Keywords:** bouldering, climbing, hangboard performance, hemodynamic response, muscle strength, vascular function

## Abstract

**Background:**

Indoor climbing can be associated with improvements in muscle strength and cardiorespiratory capacity. However, acute climbing sessions have shown to elicit an excessive hemodynamic response, and chronic vascular adaptations are still unknown. The aim of this study was to evaluate vascular adaptations and their association with grip strength and hangboard performance after an 18-week bouldering program in participants without prior climbing experience.

**Methods:**

Forearm blood flow (FBF) was measured with venous occlusion plethysmography. Muscle strength was assessed by handgrip dynamometry, muscle endurance by the dead-hang test, and body composition by air displacement plethysmography.

**Results:**

We enrolled 26 controls and 27 novice climbers. At baseline, there was no difference between controls and novice climbers regarding age, sex, body composition, FBF, muscle strength and endurance. Novice climbers showed greater delta improvement in left-hand [5.7 (IQR: −1.6–17.2) vs. −4.1 (IQR: −8.0–1.9) %; *p* = 0.001], right-hand grip strength [3.8 (IQR: −1.3–13.6) vs. −0.9 (IQR: −6.4–4.5) %; *p* = 0.011], and hangboard time [23.8 (IQR: 5.1–68.7) vs. 1.8 (IQR: −21.7–13.9) %; *p* = 0.005]. Novice climbers had better delta changes in FBF [34.0 (IQR: 2.8–54.5) vs. 0.1 (IQR: −23.1–23.6) %; *p* = 0.010] and forearm vascular conductance [26.5 (IQR: 6.7–66.7) vs. 0.3 (IQR: −23.1–20.2) %; *p* = 0.011]. A positive correlation was found between delta changes in hangboard time and forearm vascular conductance (*r* = 0.29; *p* = 0.041), along with left-hand grip strength (*r* = 0.28; *p* = 0.038).

**Conclusion:**

In conclusion, an 18-week bouldering program improved handgrip strength, hangboard time and vascular function in participants without prior climbing experience, while gains in hangboard performance were associated with improvements in vascular function.

## Introduction

Indoor climbing has traditionally been used as a valuable training tool for climbers, particularly during periods of adverse weather conditions that limited access to outdoor rock climbing. Recently, specialized climbing gyms increased in popularity worldwide, leading to structured indoor climbing routines as physical training and leisure physical activity (1). It is estimated that there are around 45 million climbers globally (2). The increase in the number of practitioners can be related to the sport's recognition as an Olympic discipline since the Tokyo Olympic Games 2020 (3).

The physical demands of indoor climbing can be associated with improvements in muscle strength and cardiorespiratory fitness capacity (4, 5), with additional benefits in chronic conditions to promote quality of life (6, 7). Moreover, recent evidence demonstrated that indoor climbing performed for recreational and leisure purposes, across a range of disciplines (i.e., lead, top rope, and boulder), achieved heart rate and duration proposed by the American College of Sport Medicine (ACSM) recommendations for cardiovascular health (8), even though a discrepancy between heart rate and oxygen consumption response during climbing has been reported (9). Indeed, climbing involves a substantial integration between anaerobic and aerobic energy systems in consequence of isometric intermittent muscle contractions (10, 11). However, acute bouldering sessions in well-trained male climbers have been shown to elicit an excessive cardiovascular response, characterized by elevated systolic blood pressure (200 ± 17 mmHg), increased diastolic blood pressure (142 ± 26 mmHg) and higher heart rate (176 ± 22 beats/min) (12). This increased cardiovascular strain during bouldering practice may be explained by a significant increase in intrathoracic pressure induced by a Valsalva-like maneuver and cumulative fatigue in the upper body that can affect the exercise pressor reflex response via type III and IV afferent fibers (12). In addition, central hemodynamic adjustments can interact with local regulatory mechanisms and contribute to determining both the magnitude and distribution of muscle blood flow. Moreover, progressive loss of muscle strength in the arms within one session in intermediate rock climbers can reach 22% and 23% in the dominant and non-dominant arm, respectively (13).

Muscle blood flow is modulated by a balance between vasoconstriction in less active regions during exercise, such as splanchnic or visceral areas, and vasodilation in more active musculature. These acute changes come from an integrative result of stimulus, including mechanical contraction effects, local metabolites production, vasodilator substances derived from endothelium and peripheral sympathetic activity attenuation within activated musculature (14, 15). Several studies using a cross-sectional design have compared vascular response of elite climbers with no-climbers, and found that elite climbers may show enhanced microcirculation, improved arterial diameter, and better capillary filtration capacity (16, 17). However, chronic vascular adaptations with indoor climbing are still unknown, particularly in bouldering and physically active untrained participants.

Therefore, the aim of this study was to evaluate vascular adaptations and its associations with grip strength and hangboard performance after an 18-week bouldering program in participants without prior climbing experience.

## Materials and methods

### Study population

This is a longitudinal experimental study conducted between February 2024 and August 2025, comprising 3 semesters, in the School of Physical Education and Sport at the University of São Paulo (EEFE-USP) and the Sports Practice Center of the University of São Paulo (CEPE-USP). Participants were eligible to participate if they had (1) between 18 and 60 years old; (2) no musculoskeletal chronic injuries or diseases that could be worsened by climbing (e.g., osteoarthritis, tendinopathies, ligament instability, stress fracture, and meniscal degeneration); and (3) no climbing experience. Participants who performed moderate-to-high intensity resistance training and/or endurance training were excluded from the study. Written informed consent was obtained from all participants before any procedure performed in the study. The experimental protocol was approved by the Ethics Research Committee of the EEFE-USP (CAAE: 76414023.0.0000.5391). The evaluation procedures took place in the EEFE-USP and climbing sessions were performed at CEPE-USP. Participants were assessed in two time points, before and after an 18-week intervention climbing program in bouldering modality. Participants were enrolled in the bouldering program through a semester course offered by EEFE-USP, which was limited to 20 spots. Enrollment followed a chronological order of registration and those selected were invited to participate in the research intervention. The other participants who showed interest in the course, but were not selected due to the number of spots, served as the control group. Therefore, randomization into the climbing training program or control group was not possible given the predetermined enrollment process. The control group was instructed to maintain their usual physical activity routines during the study. The level of physical activity was assessed by the researchers through a questionnaire with self-declaration.

**Figure 1:**
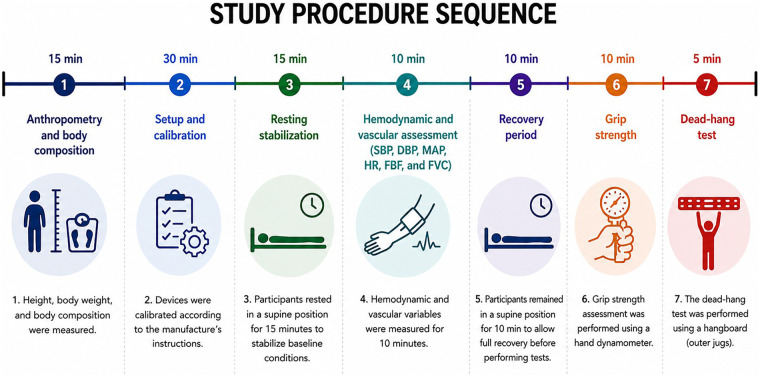
Experimental protocol performed before and after an 18-week climbing program for controls and novice climbers. SBP, systolic blood pressure; DBP, diastolic blood pressure; MAP, mean arterial pressure; HR, heart rate; FBF, forearm blood flow; FVC, forearm vascular conductance.

All assessments were conducted during a single morning laboratory visit, one week before the intervention began and within a week of its completion. Participants were refrained from caffeine and exercise 24 h before any procedure and instructed to maintain similar dietary and hydration status before testing. After anthropometric measurements, the devices were calibrated and participants were equipped. Then, participants rested for 15 min before forearm blood flow measurement (Figure 1). Grip strength assessment and dead-hang test were performed afterward. All cardiovascular measurements were conducted in a room temperature between 22 and 24 °C.

### Forearm blood flow measurements

Forearm blood flow (FBF; ml·min^−1^·100ml^−1^) was assessed using venous occlusion plethysmography technique. Forearm circumference was measured to the nearest 0.1 cm with a non-elastic measuring tape. The non-dominant arm was maintained above the level of the heart to guarantee an adequate venous drain and the participant was assessed in a resting supine position. A mercury strain gauge was placed around the widest portion of the forearm and attached to a dual channel plethysmograph (Hokanson AI6, Bellevue, WA, USA). Two pneumatic cuffs (Hokanson AI6, Bellevue, WA, USA) were positioned around the same arm, one proximal to the olecranon process and another around the wrist. The wrist cuff was inflated to a suprasystolic pressure (200 mmHg) for 30 s before beginning measurements and remained inflated throughout the assessment. The increased tension in the mercury strain gauge reflected the increasing forearm volume and, consequently, muscle blood flow measure. At 20 s intervals, the arm cuff was inflated above supravenous pressure (80–90 mmHg) for 10 s and deflated for another 10 s. The baseline FBF waveform was recorded on a polygraph and analyzed for each minute, resulting in three recordings per minute. The 10 min average analysis was used as FBF value. Systolic blood pressure (SBP) and diastolic blood pressure (DBP) were measured continuously and non-invasively on a beat-to-beat basis using a finger photoplethysmograph (Finometer Pro; Finapress Medical Systems, Amsterdam, The Netherlands). Mean arterial pressure (MAP) was calculated as MAP = [SBP+(2 x DBP)]/3. Then, forearm vascular conductance was calculated as (FBF/MAP) x 100 and expressed in arbitrary units (18). Heart rate was monitored continuously using a 5-lead electrocardiogram (ECG; AM 8200, Anamed Instruments, São Paulo, Brazil). Blood pressure, heart rate and FBF were recorded for 10 min.

### Anthropometric and muscle strength assessment

Body mass measurements were performed using air displacement plethysmography (BODPOD® body composition system; Life Measurement Instruments, CA, EUA). This method assessed body composition, as well as estimated total energy expenditure (TEE). The height of each participant was measured to the nearest 0.1 cm with a wall-mounted stadiometer. Muscle strength was assessed by a handgrip dynamometer (Model J00105; Jamar Hydraulic Hand Dynamometer, Sammons Preston Rolyan, Bolingbrook, Illinois, USA) using both hands in a neutral position with elbow flexed at 90°. There was 1 min rest interval between efforts in each hand, and the maximum value of three attempts was used (19). To assess specific muscle endurance of the forearm flexor muscles, the dead-hang test was performed using a hangboard (outer jugs). The test consists of recording the time (s) during which participants were able to hang with an open grip, arms fully extended, and knees flexed at 90° (20). After adjusting the dynamometer to fit hand size and being familiarized with the dead-hang hold, participants performed the handgrip strength and endurance tests.

### Climbing training program

The climbing training program consisted of two sessions per week, focused on bouldering techniques, and lasted for 18 weeks. Each session had 60 min divided into three parts: (1) 5–10 min warm up with specific joint movements of wrists, shoulders, hips, knees, and ankles, (2) 40–50 min of main part focusing on the tasks defined for each class (see scheduled content as Supplementary Material), and (3) 5 min of cool down. The boulder consisted mostly of vertical walls with 4-meter height and some walls had 15–30° inclination. The progression strategy during the program was based on gradually increasing physical demands of climbing, strength of forearm flexors and lower limbs through increased time spent on vertical routes, dynamic movements (power) and use of overhanging walls. The technical content of the climbing program included safety instructions, an introduction to bouldering modality, maintenance of body center mass equilibrium during climbing, proper grip techniques (e.g., crimp and pinch), hip mobility, footwork skills (e.g., toe hook, heel hook, roll on/off), crossing movements, and flag techniques. Training adherence was calculated as the number of sessions attended divided by total scheduled sessions. Training content can be checked in detail in the Supplementary Material.

### Statistical analysis

The variable results were expressed as mean ± standard deviation, median with interquartile range, or frequencies with percentage. Statistical analyses were performed using the Statistical Package for the Social Sciences version 25 for Windows (SPSS Inc., Chicago, IL, USA). A *p*-value <0.05 was used in all tests to determine the level of significance. Shapiro–Wilk test was used to verify normal distribution for all data collected. Chi-square (*χ*2) test was used to analyze categorical variables. Independent *T*-test was applied to compare baseline characteristics. Two-way repeated-measures ANOVA analysis was performed to compare parametric data for time×group interaction. When appropriate, a *post hoc* analysis of Bonferroni was applied for multiple comparisons. Changes (*Δ*) during the intervention were expressed as percentage variation, calculated by subtracting pre-test values from post-test values, dividing by the pre-test values, and multiplying by 100 [(Post−Pre)/Pre×100]. Then, Mann–Whitney test was applied and Spearman's correlations were used to correlate variables when necessary.

## Results

There was a total of 26 participants included as controls (female, *n* = 15/58%) and 27 participants as novice climbers (female, *n* = 16/59%). At baseline, there were no difference between controls and novice climbers regarding age (32 ± 11 vs. 35 ± 9 years; *p* = 0.189), height (1.66 ± 0.11 vs. 1.68 ± 0.11 m; *p* = 0.425), body mass (68.6 ± 14.3 vs. 68.5 ± 13.3 kg; *p* = 0.985), body mass index (25.1 ± 4.9 vs. 24.2 ± 3.9 kg/m²; *p* = 0.479), absolute fat mass (17.8 ± 10.4 vs. 18.0 ± 9.3 kg; *p* = 0.929), percentage fat mass (25 ± 12 vs. 26 ± 10%; *p* = 0.824), absolute fat-free mass (50.8 ± 10.9 vs. 50.5 ± 10.2 kg; *p* = 0.913), and percentage fat-free mass (75 ± 12 vs. 74 ± 10%; *p* = 0.822), respectively. Adherence to the climbing program was 90 ± 11%. The sample baseline characteristics are presented in Table 1.

**Table 1:** Baseline characteristics for controls and climbers.

Variables	Controls (*n* = 26)	Novice climbers (*n* = 27)	*P*-value
Age (years)	32 ± 11	35 ± 9	0.189
Female, *n* (%)	15 (58)	16 (59)	0.604
Body mass (kg)	68.6 ± 14.3	68.5 ± 13.3	0.985
Height (m)	1.66 ± 0.11	1.68 ± 0.11	0.425
BMI (kg/m^2^)	25.1 ± 4.9	24.2 ± 3.9	0.479
Estimated TEE (kcal/day)	2,226 ± 644	2,270 ± 593	0.788
Fat mass (kg)	17.8 ± 10.4	18.0 ± 9.3	0.929
Fat mass (%)	25 ± 12	26 ± 10	0.824
FFM (kg)	50.8 ± 10.9	50.5 ± 10.2	0.913
FFM (%)	75 ± 12	74 ± 10	0.822
Forearm circumference (cm)	25.6 ± 2.8	25.2 ± 2.5	0.563

Data are presented as mean ± standard deviation or frequencies and percentages. BMI, body mass index; FFM, fat-free mass.

At baseline, when compared controls and novice climbers there was no difference for forearm circumference (25.6 ± 2.8 vs. 25.2 ± 2.5 cm; *p* = 0.563), FBF (2.90 ± 1.43 vs. 2.36 ± 0.92 ml·min^−1^·100ml^−1^; *p* = 0.110), and forearm vascular conductance (3.64 ± 1.89 vs. 2.84 ± 1.22; *p* = 0.075). Moreover, there was no difference in heart rate (63 ± 8 vs. 65 ± 10 beats/min; *p* = 0.276), and SBP (118 ± 9 vs. 123 ± 10 mmHg; *p* = 0.058) between controls and novice climbers, whereas novice climbers showed higher DBP (65 ± 7 vs. 61 ± 6 mmHg; *p* = 0.027) and MAP (84 ± 8 vs. 80 ± 7 mmHg; *p* = 0.041) compared to controls, respectively. In addition, there was no difference in hangboard time (41 ± 22 vs. 51 ± 28 s; *p* = 0.151), right-hand (39.0 ± 10.4 vs. 40.0 ± 10.1 kg; *p* = 0.712) and left-hand grip strength (38.7 ± 9.6 vs. 39.9 ± 10.1 kg; *p* = 0.660) between controls and novice climbers, respectively (Table 2).

**Table 2:** Hemodynamic, handgrip strength and endurance variables before and after an 18-week climbing program for controls and novice climbers.

Variables	Controls (*n* = 26)		Novice climbers (*n* = 27)	
	Baseline	18 week	Baseline	18 week
Heart rate (beats/min)	63 ± 8	62 ± 9	65 ± 10	63 ± 8
SBP (mmHg)	118 ± 9	119 ± 12	123 ± 10	125 ± 19
DBP (mmHg)	61 ± 6	62 ± 8	65 ± 7*	65 ± 7
MAP (mmHg)	80 ± 7	81 ± 9	84 ± 8*	84 ± 7
FBF (ml·min^−1^·100ml^−1^)	2.90 ± 1.43	2.99 ± 1.80	2.36 ± 0.92	3.11 ± 1.31**
Forearm vascular conductance (arbitrary unit)	3.64 ± 1.89	3.87 ± 2.97	2.84 ± 1.22	3.74 ± 1.60**
Right-hand grip strength (kg)	39.0 ± 10.4	38.1 ± 9.8	40.0 ± 10.1	42.0 ± 9.3**
Left-hand grip strength (kg)	38.7 ± 9.6	37.3 ± 9.2	39.9 ± 10.1	42.2 ± 9.2*^,^**
Hangboard time (s)	41 ± 22	39 ± 21	51 ± 28	66 ± 32*^,^**

DBP, diastolic blood pressure; FBF, forearm blood flow; MAP, mean arterial pressure; SBP, systolic blood pressure.

* *p* < 0.05 compared to controls at the same timepoint.

** *p* < 0.05 compared to baseline within group.

There was a significant group×time interaction observed for FBF (*F* = 4.358; *p* = 0.042), right-hand grip strength (*F* = 4.998; *p* = 0.030), left-hand grip strength (*F* = 9.791; *p* = 0.003), and hangboard time (*F* = 15.422; *p* < 0.001).

**Figure 2:**
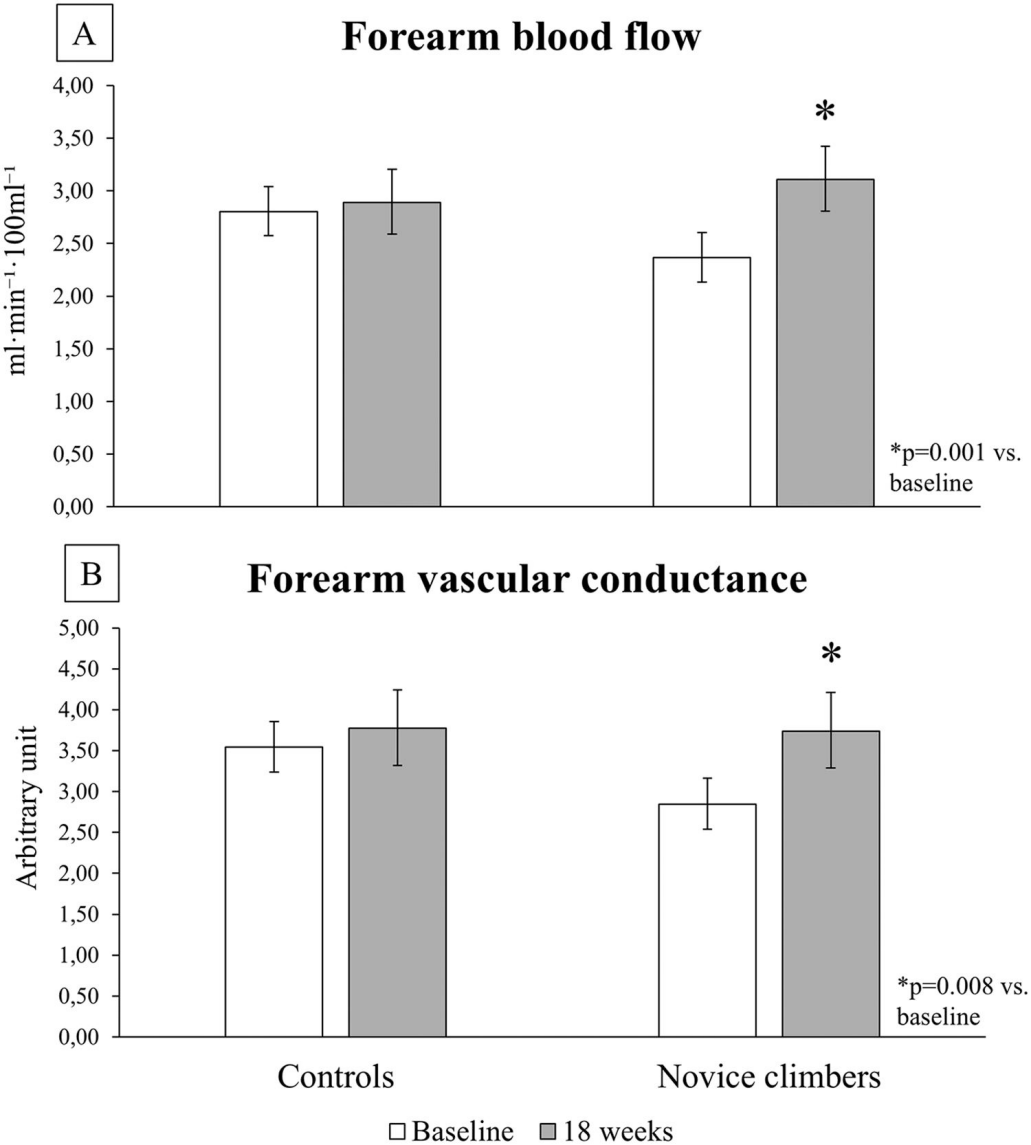
Changes in forearm blood flow **(A)** and forearm vascular conductance **(B)** after 18 weeks of bouldering training in novice climbers compared with controls.

When comparing pre- and post-intervention conditions within groups, FBF increased for novice climbers (2.36 ± 0.92 to 3.11 ± 1.31 ml·min^−1^·100ml^−1^; *p* = 0.001; Panel A/Figure 2), while controls did not improve FBF (2.90 ± 1.43 to 2.99 ± 1.80 ml·min^−1^·100ml^−1^; *p* = 0.702). Forearm vascular conductance improved for novice climbers (2.84 ± 1.22 to 3.74 ± 1.60; *p* = 0.008; Panel B/Figure 2) but not for controls (3.64 ± 1.89 to 3.87 ± 2.97; *p* = 0.490) indicating that an 18-week climbing program had a positive effect on vascular function. In addition, comparing pre- and post-intervention, novice climbers increased hangboard time (51 ± 28 to 66 ± 32 s; *p* < 0.001), right-hand (40.0 ± 10.1 to 42.0 ± 9.3 kg; *p* = 0.015) and left-hand grip strength (39.9 ± 10.1 to 42.2 ± 9.2 kg; *p* = 0.004).

At post-intervention, hangboard time (66 ± 32 vs. 39 ± 21 s; *p* < 0.001) and left-hand grip strength (42.2 ± 9.2 vs. 37.3 ± 9.2 kg; *p* = 0.043) were higher for novice climbers compared to controls (Table 2).

### Delta changes during intervention and correlations

Comparing muscle strength and hangboard changes during intervention, novice climbers showed greater percentage (*Δ*) improvement in left-hand [5.7 (IQR: −1.6–17.2) vs. −4.1 (IQR: −8.0–1.9) %; *p* = 0.001], right-hand grip strength [3.8 (IQR: −1.3–13.6) vs. −0.9 (IQR: −6.4–4.5) %; *p* = 0.011], and hangboard time [23.8 (IQR: 5.1–68.7) vs. 1.8 (IQR: −21.7–13.9) %; *p* = 0.005]. Regarding vascular function, novice climbers had better relative changes in FBF [34.0 (IQR: 2.8–54.5) vs. 0.1 (IQR: −23.1–23.6) %; *p* = 0.010] and improved forearm vascular conductance [26.5 (IQR: 6.7–66.7) vs. 0.3 (IQR: −23.1–20.2) %; *p* = 0.011] compared to controls.

A positive correlation was found between percentage changes (*Δ*) in hangboard time and forearm vascular conductance (*r* = 0.29; *p* = 0.041), as well as with left-hand grip strength (*r* = 0.28; *p* = 0.038). No significant correlations were observed between hangboard time and FBF (*r* = 0.26; *p* = 0.063) or right-hand grip strength (*r* = 0.23; *p* = 0.088). There were no correlations between both handgrip strength and vascular parameters.

## Discussion

Our findings demonstrated that an 18-week bouldering training program led to significant improvements in vascular function, evidenced by a 34% increase in FBF and a 27% enhancement in forearm vascular conductance, as well as gains in handgrip strength and hangboard performance in participants with no prior climbing experience. Moreover, changes in hangboard time were positively associated with better vascular function. Thus, these results suggest that bouldering training can induce functional and/or structural vascular adaptations in novice climbers.

Muscle perfusion during exercise is regulated by both endothelium-derived nitric oxide bioavailability (21) and endothelium-independent response related to attenuation of peripheral sympathetic drive to active musculature (22), limiting muscle blood flow to less active tissues while promoting vasodilation in the working muscles (23). It is suggested that elite climbers have a distinct structural characteristics in both peripheral resistance vessels (e.g., arteries, arterioles, and capillaries) (17) and conduit arteries (larger arteries) (24, 25) when compared to individuals who do not perform intermittent and isometric climbing movements on a chronic basis. Our findings are consistent with previous studies demonstrating improved muscle perfusion capacity, likely reflecting a reduction in vascular resistance in the forearm, probably caused by improved vasodilation and lower vasoconstriction (functional adaptation) at rest, as well as increased capillarization and arterial remodeling (structural adaptation) in novice climbers.

Previous studies evaluating forearm muscle blood flow and oxygen kinetics during intermittent and isometric climbing exercise have demonstrated increased deoxygenation during contractions, followed by enhanced reoxygenation during rest periods in climbers (24, 25). These repeated ischemia–reperfusion cycles, which increase shear stress during reperfusion phases, may act as an important stimulus for endothelial adaptation. In addition, metabolite accumulation during sustained contractions may further contribute to vasodilation and angiogenic signaling. However, this improvement in oxygen availability was not sufficient to induce chronic adaptations in vascular structure and endothelial function in experienced climbers (26). In our study, improved FBF and increased forearm vascular conductance after 18 weeks suggest that the climbing program was effective in promoting intermittent isometric contractions with substantial occlusion in forearm muscle blood flow, ultimately leading to improved muscle perfusion, oxygen delivery, and metabolite clearance at rest in novice climbers.

The ability to perfuse oxygen into the forearm flexor muscles can be a hemodynamic limitation to climbing performance at critical occluding forces (i.e., 40%–70% maximum voluntary contraction) (25); however, climbing training may enhance muscle perfusion during exercise when contractions remain below or are intermittently relieved from the critical occluding threshold. Although the significant increase in hangboard time can be attributed to the lack of experience with climbing in our sample, these adaptations may be related to an improved integration between aerobic and anaerobic energy systems required in climbing, regardless of training status and skill level of climbers (10). Thus, peripheral metabolic adaptation via afferent pressor response, promoted by increased lactate and H + ions buffer capacity, could lead to less activation of group IV afferent fibers, which reduced sympathetic drive to working muscles, causing less vasoconstriction and improving oxygen delivery and metabolites removal.

In our study, we found a positive correlation between changes in hangboard time and forearm vascular conductance, suggesting that improvements in vascular function may contribute to hangboard performance. Nevertheless, improvements in hangboard performance should not be attributed solely to enhanced muscle perfusion. Indeed, the dead-hang test can be strongly influenced by the strength-to-body-weight ratio, discomfort tolerance, and grip position reflecting a maximal isometric effort for tests with short duration. In isometric intermittent contractions during bouldering, the ability to generate high maximal force is a key determinant of performance, as increases in muscle strength reduce the relative force required to sustain a given submaximal contraction, delaying the onset of fatigue and reducing mechanical constraints on local circulation. Thus, gains in maximal force also contributed substantially to improving hangboard performance in our sample.

Upper body strength can be more important to determine climbing performance than lower body strength (27). Although handgrip strength assessed by dynamometry has been shown limited specificity in experienced climbers for discriminating performance (28), our results demonstrated a modest increase of handgrip strength (4%–5%) after an 18-week climbing program, whereas hangboard performance improved to a greater extent (24%), suggesting that a climbing specific task was more sensitive to training-induced adaptations than a grip strength measurement.

Indoor climbing can resemble cardiovascular adaptations observed during resistance training with or without blow flow restriction (29), which may elicit a larger blood pressure response due to its occlusive nature. In a study assessing intra-arterial blood pressure after two boulder problems in 6 elite climbers, it has been shown that SBP reached on average 175 ± 27 mmHg in the first boulder problem and 200 ± 17 mmHg in the second boulder problem, increasing around 40% from baseline. Moreover, DBP increased even further reaching 116 ± 19 mmHg and 142 ± 26 mmHg at peak, respectively, representing a mean increase of 60%. Although we have not assessed the acute response of blood pressure to climbing, our participants did not show an increase in resting SBP (123 ± 10 to 125 ± 19 mmHg) and DBP (65 ± 7 to 65 ± 7 mmHg) after 18 weeks of training, though climbers started with a slightly higher DBP (65 ± 7 vs. 61 ± 6 mmHg) and MAP (84 ± 8 vs. 80 ± 7 mmHg) compared to controls (Table 2), but still within a normal clinical range. These data suggest that bouldering can be hemodynamically safe to practice for healthy, young, and novice climbers.

Despite our novel findings, some limitations must be highlighted. First, our study was not randomized due to the predetermined enrollment process. Additionally, we have not assessed vascular responsiveness to specific climbing exercise, such as occlusion induced by isometric intermittent contractions. Thus, the vascular bed may respond differently when stimulated during climbing exercises. Finally, another limitation of our study is that FBF was assessed at rest and the translation of these findings to exercise conditions remains uncertain, as improvements in resting vascular tone may not necessarily reflect enhanced blood flow responses during climbing.

In conclusion, an 18-week bouldering program improved handgrip strength, hangboard time and vascular function in participants without prior climbing experience, while gains in hangboard performance were associated with improvements in vascular function.

## Data Availability

The raw data supporting the conclusions of this article will be made available by the authors, without undue reservation.

## References

[1] Mckellar BJ Coates AM Cohen JN Burr JF. Time management strategies of rock climbers in world cup bouldering finals. J Hum Kinet. (2023) 86:165–74. 10.5114/jhk/159652 37181256 PMC10170546

[2] Breen M Reed T Nishitani Y Jones M Breen HM Breen MS. Wearable and non-invasive sensors for rock climbing applications: science-based training and performance optimization. Sensors. (2023);23(11):5080. 10.3390/s23115080 37299807 PMC10255440

[3] Lutter C El-Sheikh Y Schöffl I Schöffl V. Sport climbing: medical considerations for this new Olympic discipline. Br J Sports Med. (2017) 51(1):2–3. 10.1136/bjsports-2016-096871 27821387

[4] Assmann M Steinmetz G Schilling AF Saul D. Comparison of grip strength in recreational climbers and non-climbing athletes-A cross-sectional study. Int J Environ Res Public Health. (2020) 18(1):129. 10.3390/ijerph18010129 33375452 PMC7796164

[5] Muehlbauer T Stuerchler M Granacher U. Effects of climbing on core strength and mobility in adults. Int J Sports Med. (2012) 33(6):445–51. 10.1055/s-0031-1301312 22422306

[6] Langer A Roth D Santer A Flotz A Gruber J Wizany L. Climb up! head up! climbing improves posture in Parkinson’s disease. A secondary analysis from a randomized controlled trial. Clin Rehabil. (2023) 37:2692155231174990. 10.1177/02692155231174990 PMC10492431 37157229

[7] Luttenberger K Stelzer EM Först S Schopper M Kornhuber J Book S. Indoor rock climbing (bouldering) as a new treatment for depression: study design of a waitlist-controlled randomized group pilot study and the first results. BMC Psychiatry. (2015) 15:201. 10.1186/s12888-015-0585-8 26302900 PMC4548691

[8] Smetanka RG Armenta RF Nessler JA Newcomer SC. Heart rate response, duration, grip strength, and anthropometric characteristics in recreational indoor rock climbers. J Strength Cond Res. (2022) 36(3):832–7. 10.1519/JSC.0000000000003540 35180193

[9] Callender NA Hayes TN Tiller NB. Cardiorespiratory demands of competitive rock climbing. Appl Physiol Nutr Metab. (2021) 46(2):161–8. 10.1139/apnm-2020-0566 32813982

[10] Bertuzzi RC Franchini E Kokubun E Kiss MA. Energy system contributions in indoor rock climbing. Eur J Appl Physiol. (2007) 101(3):293–300. 10.1007/s00421-007-0501-0 17602238

[11] Sheel AW Seddon N Knight A McKenzie DC R Warburton DE. Physiological responses to indoor rock-climbing and their relationship to maximal cycle ergometry. Med Sci Sports Exerc. (2003) 35(7):1225–31. 10.1249/01.MSS.0000074443.17247.05 12840646

[12] Callender NA Hart PW Ramchandani GM Chaggar PS Porter AJ Billington CP. The exercise pressor response to indoor rock climbing. J Appl Physiol. (2020) 129(2):404–9. 10.1152/japplphysiol.00357.2020 32644913

[13] Macdonald GA Manning JW Bodell NG Young JC Schilling BK Lee SP. Acute handgrip fatigue and forearm girth in recreational sport rock climbers. Int J Exerc Sci. (2022) 15(4):834–45. 10.70252/LZNO8519 35992502 PMC9362893

[14] Hearon CM Dinenno FA. Regulation of skeletal muscle blood flow during exercise in ageing humans. J Physiol. (2016) 594(8):2261–73. 10.1113/JP270593 26332887 PMC4933119

[15] Saltin B Rådegran G Koskolou MD Roach RC. Skeletal muscle blood flow in humans and its regulation during exercise. Acta Physiol Scand. (1998) 162(3):421–36. 10.1046/j.1365-201X.1998.0293e.x 9578388

[16] Thompson EB Farrow L Hunt JE Lewis MP Ferguson RA. Brachial artery characteristics and micro-vascular filtration capacity in rock climbers. Eur J Sport Sci. (2015) 15(4):296–304. 10.1080/17461391.2014.940560 25068834

[17] Ferguson RA Brown MD. Arterial blood pressure and forearm vascular conductance responses to sustained and rhythmic isometric exercise and arterial occlusion in trained rock climbers and untrained sedentary subjects. Eur J Appl Physiol Occup Physiol. (1997) 76(2):174–80. 10.1007/s004210050231 9272777

[18] Alves MJ Dos Santos MR Dias RG Akiho CA Laterza MC Rondon MU. Abnormal neurovascular control in anabolic androgenic steroids users. Med Sci Sports Exerc. (2010) 42(5):865–71. 10.1249/MSS.0b013e3181c07b74 19997008

[19] Roberts HC Denison HJ Martin HJ Patel HP Syddall H Cooper C. A review of the measurement of grip strength in clinical and epidemiological studies: towards a standardised approach. Age Ageing. (2011) 40(4):423–9. 10.1093/ageing/afr051 21624928

[20] López-Rivera E González-Badillo JJ. Comparison of the effects of three hangboard strength and endurance training programs on grip endurance in sport climbers. J Hum Kinet. (2019) 66:183–95. 10.2478/hukin-2018-0057 30988852 PMC6458579

[21] Sun H Zhang Y Shi L. Advances in exercise-induced vascular adaptation: mechanisms, models, and methods. Front Bioeng Biotechnol. (2024) 12:1370234. 10.3389/fbioe.2024.1370234 38456010 PMC10917942

[22] Goes-Santos BR Rondon E Fonseca GWP Sales ARK Santos MR Antunes-Correa LM. Physical capacity increase in patients with heart failure is associated with improvement in muscle sympathetic nerve activity. Int J Cardiol. (2023) 378:48–54. 10.1016/j.ijcard.2023.02.018 36791967

[23] Green DJ Hopman MT Padilla J Laughlin MH Thijssen DH. Vascular adaptation to exercise in humans: role of hemodynamic stimuli. Physiol Rev. (2017) 97(2):495–528. 10.1152/physrev.00014.2016 28151424 PMC5539408

[24] Fryer S Stoner L Scarrott C Lucero A Witter T Love R. Forearm oxygenation and blood flow kinetics during a sustained contraction in multiple ability groups of rock climbers. J Sports Sci. (2015) 33(5):518–26. 10.1080/02640414.2014.949828 25311579

[25] Fryer S Stoner L Lucero A Witter T Scarrott C Dickson T. Haemodynamic kinetics and intermittent finger flexor performance in rock climbers. Int J Sports Med. (2015) 36(2):137–42. 10.1055/s-0034-1385887 25251449

[26] Perrin TP Randy H Santal P Hugues X Tourette N Coudurier M. Low-Load blood flow restriction training enhances brachial blood flow during exercise but not reactive hyperemia in experienced climbers. Scand J Med Sci Sports. (2026) 36(2):e70211. 10.1111/sms.70211 41579021 PMC12831503

[27] Mermier CM Janot JM Parker DL Swan JG. Physiological and anthropometric determinants of sport climbing performance. Br J Sports Med. (2000) 34(5):359–65. 10.1136/bjsm.34.5.359 11049146 PMC1756253

[28] Watts PB Jensen RL Agena SM Majchrzak JA Schellinger RA Wubbels CS. Changes in EMG and finger force with repeated hangs from the hands in rock climbers. Int J Exerc Sci. (2008) 1(2):62–70. 10.70252/EQEO5233 27182296 PMC4739290

[29] Liu Y Jiang N Pang F Chen T. Resistance training with blood flow restriction on vascular function: a meta-analysis. Int J Sports Med. (2021) 42(7):577–87. 10.1055/a-1386-4846 33735919

