# Supplementary material for: Forearm blood flow and vascular conductance improve after 18 weeks of bouldering training in novice climbers
Source: Front Sports Act Living. 2026 Jun 1;8:1818579. doi: 10.3389/fspor.2026.1818579 (PMC13267177; doi:10.3389/fspor.2026.1818579)
Supplement: Supplementary file 1 [file Table1.docx]

**Supplementary material**

**Table 1.** Climbing program.

| **Week** | **Session 1** | **Session 2** |
| --- | --- | --- |
| **1** | Introduction to bouldering modality, explanation of the routes difficulty level, description of starting and finishing rules, free climbing, and training for falling safely on the mattress (absorbing the impact during falling with lower limbs while performing an appropriate squatting movement, crossing the arms on the chest and transferring weight backwards to fall lying down). | Free climbing without a previous route, training descending climbing, and training for falling safely on the mattress. |
| **2** | Explanation and demonstration of how to hold different types of holds and how to apply force with the arm extended perpendicular to the base of a hold, forming a “T” shape between the base of the grip and the arm. | Explanation and demonstration of how to hold different types of holds and how to apply force with the arm extended perpendicular to the base of a hold, forming a “T” shape between the base of the grip and the arm. |
| **3** | Explanation and demonstration of how to position and apply force on the base of the holds with the tips of the toes. | Explanation and demonstration of how to position and apply force on the base of the holds with the tips of the toes. |
| **4** | Ladder movement training on climbing walls, applying alternate force between opposite upper and lower limbs (e.g., left hand and right foot followed by right hand and left foot). | Ladder movement training on climbing walls, applying alternate force between opposite upper and lower limbs (e.g., left hand and right foot followed by right hand and left foot). |
| **5** | Development of the unilateral or bilateral hip-opening technique to allow a closer, parallel positioning to the wall. This technique can be performed by combining hip flexion with abduction, in a “frog” position. | Development of the unilateral or bilateral hip-opening technique to allow a closer, parallel positioning to the wall. This technique can be performed by combining hip flexion with abduction, in a “frog” position. |
| **6** | Differentiation between ladder-style climbing movements (practiced since week 4) and crossed “X” movements, applying diagonal force with hips parallel to the wall (e.g., right foot with left hand and left foot with right hand). This position can be achieved by combining hip flexion with adduction. | Differentiation between ladder-style climbing movements (practiced since week 4) and crossed “X” movements, applying diagonal force with hips parallel to the wall (e.g., right foot with left hand and left foot with right hand). This position can be achieved by combining hip flexion with adduction. |
| **7** | Crossed “X” movement training - 1x2 (forming a triangle with one hand and two feet), consisting of one hand movement and two foot movements. For example, starting with left hand together with right foot and left foot (triangle 1), followed by right hand with left foot and right foot (triangle 2), and so on. | Crossed “X” movement training - 1x2 (forming a triangle with one hand and two feet), consisting of one hand movement and two foot movements. For example, starting with left hand together with right foot and left foot (triangle 1), followed by right hand with left foot and right foot (triangle 2), and so on. |
| **8** | Crossed “X” movement training - 1x2 (forming a triangle with one hand and two feet), consisting of one hand movement and two foot movements. For example, starting with left hand together with right foot and left foot (triangle 1), followed by right hand with left foot and right foot (triangle 2), and so on. | Crossed “X” movement training - 1x2 (forming a triangle with one hand and two feet), consisting of one hand movement and two foot movements. For example, starting with left hand together with right foot and left foot (triangle 1), followed by right hand with left foot and right foot (triangle 2), and so on. |
| **9** | Development of the outside flag technique, with one foot positioned on the hold and the other foot unsupported, applying pressure against the wall. | Development of the outside flag technique, with one foot positioned on the hold and the other foot unsupported, applying pressure against the wall. |
| **10** | Development of the back flag technique, with one foot on a hold and the other foot unsupported behind the supporting leg, while applying pressure against the wall or during a pendulum movement. | Development of the back flag technique, with one foot on a hold and the other foot unsupported behind the supporting leg, while applying pressure against the wall or during a pendulum movement. |
| **11** | On walls with different inclinations, training of the 1x2 crossed “X” movement as performed on week 7 and 8. | On walls with different inclinations, training of the 1x2 crossed “X” movement as performed on week 7 and 8. |
| **12** | On walls with different inclinations, training of the 1x2 crossed “X” movement as performed on week 7 and 8. | On walls with different inclinations, training of the 1x2 crossed “X” movement as performed on week 7 and 8. |
| **13** | Development of the inside flag technique, with one foot on a hold, hand on the side on another hold, and the unsupported foot crossing inside accompanied by trunk/hip rotation toward the wall. This technique is used to avoid switching feet on the same hold. | Development of the inside flag technique, with one foot on a hold, hand on the side on another hold, and the unsupported foot crossing inside accompanied by trunk/hip rotation toward the wall. This technique is used to avoid switching feet on the same hold. |
| **14** | Development of foot-switching techniques on the same hold: roll on/roll off and cover/remove. These techniques consist of one foot over the other or next to the other during weight transfer. The foot supported on the hold will make a weight transfer to the foot that is coming from above (cover/remove) or laterally (roll on/roll off). | Development of foot-switching techniques on the same hold: roll on/roll off and cover/remove. These techniques consist of one foot over the other or next to the other during weight transfer. The foot supported on the hold will make a weight transfer to the foot that is coming from above (cover/remove) or laterally (roll on/roll off). |
| **15** | Traverse training with horizontal movements on the wall, avoiding minimal vertical movement. To encourage lateral movements by applying techniques learned from previous training sessions. | Traverse training with horizontal movements on the wall, avoiding minimal vertical movement. To encourage lateral movements by applying techniques learned from previous training sessions. |
| **16** | Development of heel and toe hook, instead of using the tip of the toes. These techniques allow for more stable movements during climbing, engaging hamstrings. | Development of heel and toe hook, instead of using the tip of the toes. These techniques allow for more stable movements during climbing, engaging hamstrings. |
| **17** | Training of different hand-grip techniques (pinch and crimp). These techniques are applied on specific holds. | Training of different hand-grip techniques (pinch and crimp). These techniques are applied on specific holds. |
| **18** | Free climbing applying all techniques taught throughout the course. | Free climbing applying all techniques taught throughout the course. |
